# Causal influence of muscle weakness on cardiometabolic diseases and osteoporosis

**DOI:** 10.1038/s41598-023-46837-y

**Published:** 2023-11-15

**Authors:** Xiaoqing Mou, Bin He, Muzi Zhang, Yong Zhu, Yunsheng Ou, Xiaojun Chen

**Affiliations:** 1 Department of Radiology, The Affiliated Hospital of Southwest Medical University, Luzhou, Sichuan China; 2 Department of Orthopedics, The First Affiliated Hospital of Chongqing Medical University, Chongqing, China; 3 Department of Orthopedics, The Affiliated Traditional Chinese Medicine Hospital of Southwest Medical University, Luzhou, Sichuan China

**Keywords:** Diseases, Endocrinology, Medical research

## Abstract

The causal roles of muscle weakness in cardiometabolic diseases and osteoporosis remain elusive. This two-sample Mendelian randomization (MR) study aims to explore the causal roles of muscle weakness in the risk of cardiometabolic diseases and osteoporosis. 15 single nucleotide polymorphisms (SNPs, P < 5 × 10^−8^) associated with muscle weakness were used as instrumental variables. Genetic predisposition to muscle weakness led to increased risk of coronary artery disease (inverse variance weighted [IVW] analysis, beta-estimate: 0.095, 95% confidence interval [CI]: 0.023 to 0.166, standard error [SE]:0.036, P-value = 0.009) and reduced risk of heart failure (weight median analysis, beta-estimate: − 0.137, 95% CI − 0.264 to − 0.009, SE:0.065, P-value = 0.036). In addition, muscle weakness may reduce the estimated bone mineral density (eBMD, weight median analysis, beta-estimate: − 0.059, 95% CI − 0.110 to − 0.008, SE:0.026, P-value = 0.023). We found no MR associations between muscle weakness and atrial fibrillation, type 2 diabetes or fracture. This study provides robust evidence that muscle weakness is causally associated with the incidence of coronary artery disease and heart failure, which may provide new insight to prevent and treat these two cardiometabolic diseases.

## Introduction

Muscle weakness commonly occurs as the advancing age and it is a fundamental component of frailty and sarcopenia^1–3^. Compared to individuals in twenties, population with over 70 years are estimated to suffer from up to 20% lost muscle mass^4^. Loss of muscle mass (sarcopenia) is closely associated with muscle weakness which may affect health outcomes^5,6^. Patients with muscle weakness commonly have some difficulties in daily activity and low muscle strength as measured by hand grip strength, which has become a predictive factor of morbidity and mortality^4,7^. Muscle weakness is heritable and can be used for genetic studies^8^.

Several observational studies reported that muscle weakness had some association with the incidence of cardiometabolic diseases and osteoporosis, but these results are conflicting^9–15^. Potential confounding factors and reverse causality in these studies may affect the association between muscle weakness and cardiometabolic diseases/osteoporosis. Cardiometabolic diseases and osteoporosis are also highly polygenic traits based on the results of genome-wide association studies (GWASs)^16–22^.

Mendelian randomization (MR) study is widely used to establish the causal relationship between exposure phenotype and outcome phenotype, with the advantages of preventing reverse causation and potential confounding factors^23–27^. Furthermore, the two-sample MR study is able to increase the scope and statistical power of MR^25,28–31^. Due to the high heritability of muscle weakness, cardiometabolic diseases and osteoporosis, this two-sample MR study aims to explore the causal influence of muscle weakness on the incidence of cardiometabolic diseases and osteoporosis.

## Methods

### Genetic instrument for muscle weakness

The largest available GWAS meta-analysis included 22 independent cohorts with maximum hand grip strength recorded (i.e. the UK Biobank, the US Health and Retirement Study, the Framingham Heart Study, and others) and total 256,523 individuals of European descent aged 60 years or older. Among them, 46,596 participants was diagnosed with muscle weakness based on hand grip strength and EWGSOP definition: grip strength < 30 kg for male individuals and < 20 kg for female individuals^32^.

Initially, 15 single nucleotide polymorphisms (SNPs) showed robust association with muscle weakness (P < 5 × 10^−8^). Linkage disequilibrium (LD) between selected SNPs was calculated using European samples from the 1000 Genomes project. No SNPs were excluded due to high LD (r^2^ ≥ 0.001). Finally, 15 SNPs were used as instrumental variables (Supplementary Table 1). The proxy SNPs in linkage disequilibrium (LD, r^2^ > 0.9) were used if original SNPs were unavailable in the outcome database. Thus, rs6488725 was used as the proxy for rs34464763 among all outcomes (Supplementary Table 2).

### Outcome data sources

The genetic associations of each outcome from GWASs were presented in Table 1. Briefly, we included the GWAS summary data of cardiometabolic diseases including coronary artery disease (547,261 individuals) from UK Biobank and CARDIoGRAMplusC4D^33^, heart failure (977,323 individuals) from UK Biobank^34^, atrial fibrillation (587,446 individuals) from one large meta-analysis^35^ and type 2 diabetes (898,130 individuals) from DIAGRAM^36^. In terms of osteoporosis and fracture, the outcome measures included bone mineral density (BMD) as estimated by heel quantitative ultrasound (eBMD) and fracture among 426,824 people. Fracture cases were defined as any fracture apart from the fracture of skull, face, hands, feet, pathological fractures due to malignancy, atypical femoral fractures, periprosthetic and healed fracture^37^. Most GWASs were adjusted for sex, body mass index (BMI) and genetic principal components. All participants were all from European descent except for those with atrial fibrillation from predominant European descent (mixed descents). Supplementary Table 2 showed the summary statistics for the SNPs related to muscle weakness and corresponding statistics of outcomes.

**Table 1:** Details of studies and datasets used for analyses.

	Traits	Samples size	Population	Consortium or cohort study (link URL)
Exposure	Muscle weakness	2,56,523	European	Meta-analysis of 22 cohorts
Cardiometabolic diseases	Coronary artery disease	5,47,261	European	UK Biobank and CARDIoGRAMplusC4D (https://cvd.hugeamp.org/)
	Heart failure	9,77,323	European	UK Biobank (http://www.broadcvdi.org/)
	Atrial fibrillation	5,87,446	Mixed	Meta analysis of more than 50 studies (http://www.broadcvdi.org/)
	Type 2 diabetes	8,98,130	European	DIAGRAM (http://diagram-consortium.org)
Osteoporosis	eBMD	4,26,824	European	GEFOS (http://www.gefos.org)
	Fracutre	4,26,824	European	

### Statistical analyses

To determine causal influence of muscle weakness on each outcome, we conducted the inverse variance weighted (IVW) analysis because more than 2 SNPs were available. IVW method used a meta-analysis approach to combine Wald estimates for each SNP in order to get the overall estimates of the effect of muscle weakness on each outcome^23^. The weighted median and MR-Egger regression methods were also applied to estimate the effects. Cochrane’s Q-statistic was used to assess the heterogeneity of SNP effects and P < 0.05 indicated significant heterogeneity^38^. MR pleiotropy residual sum and outlier test (MR-PRESSO) aimed to assess the presence of pleiotropy and the effect estimates were recalculated after outlying SNPs were excluded^39^.

All methods were carried out in accordance with relevant guidelines and regulations. All experimental protocols were approved and the ethical approval for each study can be found in the original publications (including informed consent from each participant). P < 0.05 indicated statistical difference. All of these analyses were conducted in R V.4.0.4 by using the R packages of ‘MendelianRandomization’^40^, ‘TwoSampleMR’^41^ and ‘MR-PRESSO’^42^.

### Ethical approval

The ethical approval for each study included in this investigation can be found in the original publications.

## Results

### Cardiometabolic diseases

**Figure 1:**
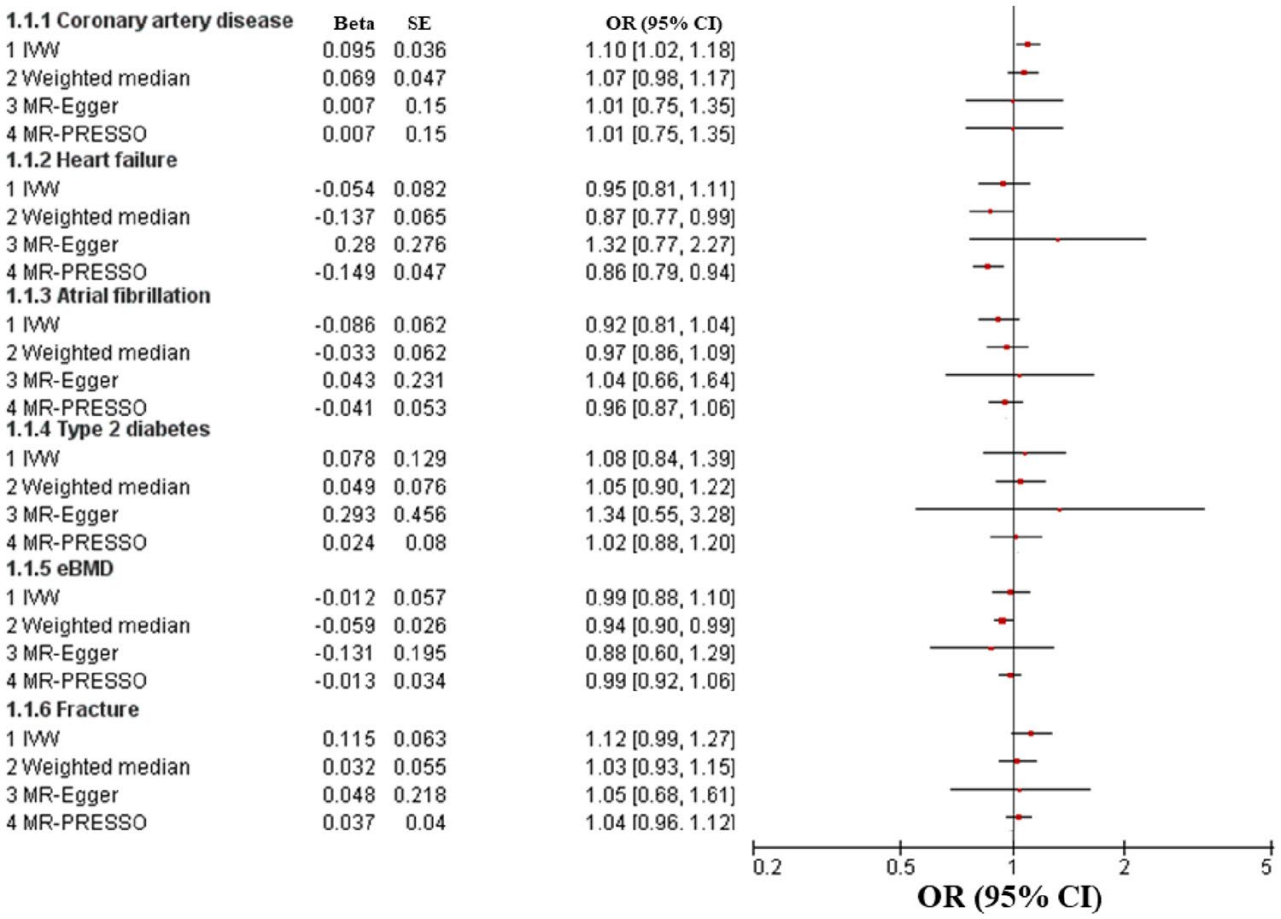
OR (95% CI) for causal association between muscle weakness and each outcome through multiple analyses.

We evaluated the causal effect of muscle weakness on coronary artery disease, heart failure, atrial fibrillation and type 2 diabetes in this MR analysis (Table 2). IVW analysis demonstrated that genetically muscle weakness played a significant causal role in the increased risk of coronary artery disease (beta-estimate: 0.095, 95% CI 0.023 to 0.166, SE:0.036, P-value = 0.009), but it was not supported by the weighted-median analysis (beta-estimate: 0.069, 95% CI − 0.023 to 0.161, SE:0.047, P-value = 0.141, Fig. 1).

**Table 2:** Mendelian randomization estimates of muscle weakness on outcomes.

Variables	IVW							Weighted median				MR-Egger							
	Estimate	SE	95% CI	P-value	Q value	I^2^	Heterogeneity P value	Estimate	SE	95% CI	P-value	Estimate	SE	95% CI	P-value	Intercept	SE	95% CI	Pleiotropy P value
Cardiometabolic disease																			
Coronary artery disease	0.095	0.036	0.023, 0.166	0.009	15.716	17.30%	0.265	0.069	0.047	− 0.023, 0.161	0.141	0.007	0.150	− 0.287, 0.300	0.965	0.005	0.008	− 0.011, 0.020	0.545
Heart failure	− 0.054	0.082	− 0.214, 0.106	0.506	47.308	70.40%	0.000	− 0.137	0.065	− 0.264, − 0.009	0.036	0.280	0.276	− 0.261, 0.821	0.310	− 0.019	0.015	− 0.048, 0.010	0.206
Atrial fibrillation	− 0.086	0.062	− 0.207, 0.035	0.162	30.186	53.60%	0.007	− 0.033	0.062	− 0.154, 0.088	0.595	0.043	0.231	− 0.411, 0.496	0.853	− 0.007	0.012	− 0.031, 0.017	0.562
Type 2 diabetes	0.078	0.129	− 0.175, 0.331	0.547	130.963	89.30%	0.000	0.049	0.076	− 0.101, 0.198	0.523	0.293	0.456	−0. 600, 1.186	0.520	− 0.012	0.025	−0. 060, 0.036	0.621
Osteoporosis																			
eBMD	− 0.012	0.057	− 0.123, 0.099	0.837	435.276	96.80%	0.000	−0. 059	0.026	− 0.110, − 0.008	0.023	− 0.131	0.195	− 0.513, 0.251	0.502	0.007	0.011	− 0.014, 0.028	0.522
Fracture	0.115	0.063	−0. 008, 0.238	0.066	42.444	67.00%	0.000	0.032	0.055	− 0.075, 0.139	0.561	0.048	0.218	− 0.380, 0.476	0.827	0.004	0.012	−0. 020, 0.027	0.745

According to weighted-median analysis, muscle weakness showed substantially causal effect on the reduced incidence of heart failure (beta-estimate: − 0.137, 95% CI − 0.264 to − 0.009, SE:0.065, P-value = 0.036), but it was not confirmed in the IVW analysis (beta-estimate: − 0.054, 95% CI − 0.214 to 0.106, SE:0.082, P-value = 0.506, Fig. 1). In addition, IVW analyses found that muscle weakness demonstrated no remarkable MR association with atrial fibrillation (beta-estimate: − 0.086, 95% CI − 0.207 to 0.035, SE:0.062, P-value = 0.162) or type 2 diabetes (beta-estimate: 0.078, 95% CI − 0.175 to 0.331, SE:0.129, P-value = 0.547), which were also confirmed by weighted-median analyses (Fig. 1).

### Osteoporosis

This MR analysis also included outcome measures of eBMD and fracture (Table 2). According to weighted-median analysis, muscle weakness was casually associated with decreased eBMD (beta-estimate: − 0.059, 95% CI − 0.110 to − 0.008, SE:0.026, P-value = 0.023), but it was not supported by the IVW analysis (beta-estimate: − 0.012, 95% CI − 0.123 to 0.099, SE:0.057, P-value = 0.827, Fig. 1). Muscle weakness revealed no causal influence on fracture by IVW analysis (beta-estimate: 0.115, 95% CI − 0.008 to 0.238, SE:0.063, P-value = 0.066) or weighted-median analysis (beta-estimate: 0.032, 95% CI − 0.075 to 0.139, SE:0.055, P-value = 0.561, Fig. 1).

### Evaluation of assumptions and sensitivity analyses

Little evidence of directional pleiotropy was found for all models (MR-Egger intercept P-value > 0.05, Table 2). There was significant heterogeneity for heart failure, atrial fibrillation, type 2 diabetes, eBMD and fracture. Thus, among the 15 SNP instrumental variables associated with muscle weakness, MR-PRESSO method identified 2 outliers (rs13107325, rs10952289) for heart failure, one outlier (rs143384) for atrial fibrillation, four outliers (rs7624084, rs34415150, rs10952289, rs62102286) for type 2 diabetes, 11 outliers (rs12140813, rs958685, rs7624084, rs13107325, rs34415150, rs10952289, rs11236213, rs34464763, rs3118903, rs8061064, rs62102286) for eBMD and 2 outliers (rs10952289, rs34464763) fracture (Table 3).

**Table 3:** Mendelian randomization estimates between muscle weakness and outcomes after excluding outliers detected by MR-PRESSO.

Outcomes	Estimate	SE	95% CI	P-value
Heart failure excluding 2 outliers (rs13107325, rs10952289)	− 0.149	0.047	−0. 241, −0. 056	0.002
Atrial fibrillation excluding one outlier (rs143384)	− 0.041	0.053	− 0.144, 0.063	0.441
Type 2 diabetes excluding four outliers (rs7624084, rs34415150, rs10952289, rs62102286)	0.024	0.080	− 0.131, 0.180	0.759
eBMD excluding 11 outliers (rs12140813, rs958685, rs7624084, rs13107325, rs34415150, rs10952289, rs11236213, rs34464763, rs3118903, rs8061064, rs62102286)	− 0.013	0.034	− 0.079, 0.053	0.704
Fracture excluding 2 outliers (rs10952289, rs34464763)	0.037	0.040	− 0.042, 0.117	0.355

After excluding these outlying SNP variants, these remarkable MR associations were confirmed between muscle weakness and increased risk of coronary artery disease (Fig. 1 and Table 3). In addition, muscle weakness was confirmed to have a causal effect on low risk of heart failure (beta-estimate: − 0.149, 95% CI − 0.241 to − 0.056, SE:0.047, P-value = 0.002, Fig. 1 and Table 3). The MR association between muscle weakness with other outcomes were not changed after excluding the outlying SNP variants (Table 3).

## Discussion

Our two-sample MR study found the robustly causal effect of muscle weakness on increased risk of coronary artery disease and decreased risk of heart failure, and these strong MR associations were confirmed by the sensitivity analyses. These positive findings indicated that the regulatory mechanisms of muscle weakness may provide new insight to prevent and treat coronary artery disease and heart failure. In addition, muscle weakness may have a causal role in reduced eBMD. We found no causal effect of muscle weakness on atrial fibrillation, type 2 diabetes or fracture.

Several observational studies and meta-analysis explored the association between muscle results and cardiometabolic diseases, but no conclusive results were found^9,13,43^. One meta-analysis revealed that handgrip strength was an independent predictor of cardiometabolic diseases in community-dwelling populations, but this this association was not significant after adjusting for baseline risk factors^9,43^. One recent MR analysis found no causality in the association between handgrip strength (European population) and coronary artery disease (mixed population). The large-scale genetic discovery analysis identified 16 loci associated with grip strength (P < 5 × 10^−8^) among 195,180 individuals as instrumental variables^10^, and that MR study included GWAS summary data related to coronary heart disease among 184,305 individuals^44^.

Our large-scale MR study was performed in larger populations including 256,523 individuals of European descent for muscle weakness and 547,261 individuals of European descent for coronary artery disease. Totally, 15 loci associated with grip strength (P < 5 × 10^−8^) were used as instrumental variables. The results provided the robust evidence for the causal association between muscle weakness and increased risk of coronary artery disease, which was confirmed by multiple sensitivity analyses. Muscle weakness and low muscle mass reduces total energy expenditure, which may result in high fat mass. Accumulated body fat mass triggers chronic inflammation, and is thought to be a risk factor for the development and progression of coronary artery disease^45–47^.

One leading cause of heart failure is coronary artery disease, but heart failure can be also caused by arrhythmias, hypertension, type 2 diabetes mellitus, obesity, and lifestyle factors (such as smoking). A large-scale observational study found that higher hand grip strength was independently associated with lower incidence of heart failure^48^. On the contrary, our MR study revealed that muscle weakness was causally associated with lower incidence of heart failure, which was confirm by the IVW analysis after excluding the outlying SNPs (beta-estimate: − 0.149, 95% CI − 0.241 to − 0.056, SE:0.047, P-value = 0.002, Fig. 1 and Table 3). This positive finding was very interesting, and may be attributed by the atrophy of the muscle fibers and reduced requirement of cardiac output due to low muscle mass^49^.

Patients with osteoporosis typically have the features of low bone mass, BMD and bone strength, which can increase the risk of fracture^50–54^. Several observational studies revealed the significant correlation between low grip strength and low BMD of the bones adjacent to the muscles related to grip^55–57^. In 1,168 menopausal women, Osei-Hyiaman et al. found the significant relationship between grip strength and BMD of metacarpal index^55^. Hasegawa et al. revealed that BMD of the distal radius was more associated with hand grip strength than with cross-sectional muscle area^57^. In contrast, Zimmermann et al. documented that hand grip strength in postmenopausal women showed no impact on vertebral BMD, but only affected femur BMD^58^, while Foley et al. documented no correlation between hand grip strength and femoral BMD^59^.

Considering these insistent results, our MR analyses revealed that muscle weakness may have a causal role in reduced eBMD. It is postulated that muscle contraction force provides a mechanical stress on the bones, which is accepted as an important osteogenic stimulus. There is bi-directional bone-muscle crosstalk, which is probably mediated by cytokines, osteokines, myokines, and other growth factors^60^. In addition, low BMD associated with muscle weakness may be associated with systemic inflammation and oxidative stress^61,62^.

Our results demonstrated that genetically muscle weakness was unlikely to be causally associated with atrial fibrillation, type 2 diabetes or fracture. The potential causal effect of muscle weakness to reduce eBMD was not translated to affect the risk of fracture. This two-sample MR study aims to investigate the causal effect of muscle weakness on the risk of cardiometabolic diseases and osteoporosis, and has the advantage of preventing reverse causation and confounding factors. The intercepts for the MR-Egger analysis suggest no directional pleiotropy for all outcomes. However, several limitations should be taken into consideration. Firstly, all the included participants are of predominantly European, and we can not directly apply our findings for other populations. Secondly, GWAS summary statistics can not be used to conduct MR analysis based on different age stratums. Thirdly, the contribution of muscle weakness to low eBMD is not translated to increased incidence of fracture, but the detail mechanisms are unclear.

## Conclusion

This two-sample MR study provides strong evidence to confirm that muscle weakness is a significantly causal factor for increased risk of coronary artery disease and reduced risk of heart failure, and the related mechanisms may help prevent and treat these two diseases.

### Supplementary Information

Supplementary Tables.

## Data Availability

Data supporting the findings of this study were available within the paper.
